# Factors associated with the death of healthcare workers due to COVID-19 in the state of Amapá, Brazil

**DOI:** 10.47626/1679-4435-2022-911

**Published:** 2022-03-30

**Authors:** Arthur Arantes Cunha, Rodolfo Antonio Corona, João Silvestre Silva-Junior, Emerson Augusto Castilho-Martins

**Affiliations:** 1 Departamento de Ciências Biológicas e da Saúde, Curso de Medicina, Universidade Federal do Amapá (Unifap), Macapá, AP, Brazil.; 2 Departamento de Medicina, Centro Universitário São Camilo, São Paulo, SP, Brazil.; 3 Programa de Pós-Graduação em Ciências da Saúde, Departamento de Pós-Graduação, Unifap, Macapá, AP, Brazil.; 4 Programa de Pós-Graduação em Ciências Ambientais, Departamento de Pós-Graduação, Unifap, AP, Brazil.

**Keywords:** healthcare workers, coronavirus infections, occupational health, epidemiology, regression analysis

## Abstract

**Introduction:**

Frontline healthcare workers providing care for COVID-19 are more likely to get infected and die compared with other professionals. Deaths or sick leaves due to COVID-19 can affect the smooth operation of health services in areas with shortage of workers.

**Objectives:**

To analyze factors associated with the death of healthcare workers due to COVID-19 in the state of Amapá, Brazil.

**Methods:**

Analytical cross-sectional study using COVID-19 data from Amapá between March 2020 and January 2021. The association of independent variables (sex, race/color, age group, region of residence, comorbidity) with death was analyzed by logistic regression.

**Results:**

Data from 1,258 workers were analyzed. The majority were women (67.7%; 852/1,258), multiracial (66.9%; 759/1,135), aged between 18 and 64 (98.3%; 1,226/1,247), with no comorbidity (86.6%; 1,090/1,258), from the Macapá metropolitan area (56.7%; 713/1,258). The mortality rate was 1.59%. Factors associated with death were: age group = 65 years (odds ratio = 10.43; 95% confidence interval [CI] = 2.78-39.11), comorbidity (odds ratio = 4.52; 95%CI = 1.74-11.74), and residence in the Macapá metropolitan area (odds ratio = 4.37; 95%CI = 1.25-15.29).

**Conclusions:**

The recognition of factors that may have caused the death of healthcare workers in Amapá can support the recommendation of protective measures for the most susceptible, such as switching to activities with lower exposure to the virus or teleworking.

## Introduction

Frontline healthcare workers providing care for suspected and confirmed cases of COVID-19, caused by severe acute respiratory syndrome coronavirus 2 (SARS-CoV-2), have increased chance of infection.^1-3^ A retrospective cohort study with Chinese healthcare workers showed an increased risk of infection in individuals with a history of suspected exposure or who provide care to patients with COVID-19, increasing workload, and improper use of personal protective equipment (PPE).^3^ Another prospective cohort study with English healthcare workers showed a high risk of developing severe symptoms of the disease when compared to other professional groups.^4^ These factors, as well as shortage of tests, are challenging for the safety and health management of these workers in Brazil.^5,6^

Absenteeism due to COVID-19 can affect the operation of health services,^2,5^ especially in areas with lower rates of healthcare workers per inhabitant, such as the Northern region of Brazil.^7^ The state of Amapá has a history of low density of technicians and scientific professionals and poor medical and hospital infrastructure,^8,9^ further aggravated by only about 16% of the local physicians work in inland municipalities.^7^ This shortfall of healthcare workers hinders the management of the pandemic in these municipalities, as they are affected by the high number of healthcare workers withdrawing from facilities during the pandemic,^10^ especially when considering the assistance to Indigenous people concentrated in inland areas.^11^

In this regard, it is significant that Amapá had the highest cumulative mortality rate (0.67/1,000) of nursing professionals due to COVID-19 in the country between the 12th and 22nd epidemiological week of 2020, a number four times higher than that in the state of Acre, with the second highest rate (0.17/1,000) in the country.^10^ Furthermore, Amapá had one of the highest prevalence of antibodies to SARS-CoV-2 in Brazil.^12^ A second wave of deaths hit Amapá from November 2020 to April 2021, showing no sign of reducing mortality rate.^13^ Some of the main consequences of the pandemic were economic loss with social impacts on the population, and the overload of healthcare services, due to the high occupancy of beds and sick leave of healthcare workers affected by COVID-19.^13^

Guerrero-Torres et al.^2^ stands out among studies that analyzed factors associated with the death of healthcare workers due to COVID-19. They found that older, male physicians with comorbidities were factors associated with the death of Mexican healthcare workers.^2^ Duprat and Melo,^10^ in a study with nursing professionals in Northern Brazil, reported that male, older individuals were more susceptible to death. However, this study was limited to the analysis of these three variables and did not include all healthcare workers.^10^

This study aimed to analyze the factors associated with death of healthcare workers due to COVID-19 in the state of Amapá, Brazil.

## Methods

### Study site

The state of Amapá is located in the Brazilian Amazon, on the left bank of the Amazon River, in the Northern region of Brazil, and it has 16 municipalities. In 2020, it had a population of 862,000 people and a population density of 6.05 inhabitants/km^2^. Macapá (capital city), Santana, and Mazagão comprise the metropolitan area of Macapá (MMA), which houses approximately 75% of the population. It has a demographic density of 30.75 inhabitants/km^2^, and a relevant socio-spatial segregation.^11,14^ According to data from the 2010 Demographic Census, Amapá had a Human Development Index of 0.708, the median per capita income was R$ 213.26 (the minimum wage was R$ 510.00 in 2010), and 25% of the population was considered poor.^8,9,11^ Historically, Amapá faces major healthcare issues, with insufficient capillarity of policies, services, and medical professionals, as well as limited and unevenly distributed hospital infrastructure.^7,9,14^ Among Brazilian capital cities, Macapá had the lowest number of health facilities in 2017 and the lowest rate of physicians per inhabitant.^7,8^

### Study design and data source/variables

Analytical cross-sectional study with analysis of secondary public data extracted from the COVID-19 Panel, an online platform developed by the Amapá Department of Health (SESA) and available on the Internet.^13^ The information used in this study were entered in the database between March 22, 2020, and January 23, 2021. The events of interest in this study were death of healthcare workers due to COVID-19 who lived in Amapá, Brazil.

This study includes data recorded in the SESA database from healthcare workers with a confirmed diagnosis of COVID-19 (rapid antibody test, rapid antigen test, or real-time polymerase chain reaction), residing in Amapá, and a clinical outcome of death or cure. The data of the individuals included in the analysis were sorted based on a dichotomous (yes/no) field referring to the healthcare workers. Data did not include empty, ignored, or fields filled in as no. Cases with unspecified clinical outcome of cure or death were also excluded because these could be active cases of the disease.

The variables were categorized as follows: “clinical outcome” (death; cure); “sex” (male; female); “race/color” (black; multiracial; white; indigenous; yellow); the variable “age group” was adapted, changing from the individual age to age ranges (aged between 18 and 64; 65 years or older); the “region of residence of the individual” was adapted from the presentation in the database, being categorized as MMA or other municipalities; and the variable “comorbidity” was transformed into a dichotomous variable (yes; no) due to the several possibilities of filling out this field in the database.

### Data analysis

The dependent variable in all analyses was the clinical outcome. The association of independent variables with the clinical outcome of death or cure was initially analyzed using the chi-square (*χ*^2^) test for independence or Fisher’s exact test. This first part of the analysis, which aims to identify variables with the potential to be included in the regression analysis, considered variables with a significance level of p-value ≤ 0.20.^15^ The selected variables were submitted to univariate logistic regression analysis. Then, we constructed the multiple model, and the independent variables were entered according to the increasing order of p-value in the univariate regression.

The final multiple model was defined using the Omnibus Tests of Model Coefficients, the Hosmer-Lemeshow test, the C statistic (area under the receiver operating characteristic curve [ROC]), and the analysis of the potential confounding of independent variables. The model was built using the forward method and “forced entry” to check for possible adjustment variables. Multicollinearity analysis was based on the inflationary variance factor^15^. The regression analyses had odds ratio (OR) and 95% confidence interval (95%CI), and the significance level p-value ≤ 0.05 was used in the Wald test. Statistical Package for the Social Sciences^®^ version 20.0 was used for analyses.

### Ethical issues

This study used a secondary, public domain, open access database. The data used did not allow individual identification. Thus, this study was exempt from evaluation by an ethics committee for research in human beings according to the Brazilian Ethical Standards of Scientific Research.

## Results

**Figure 1:**
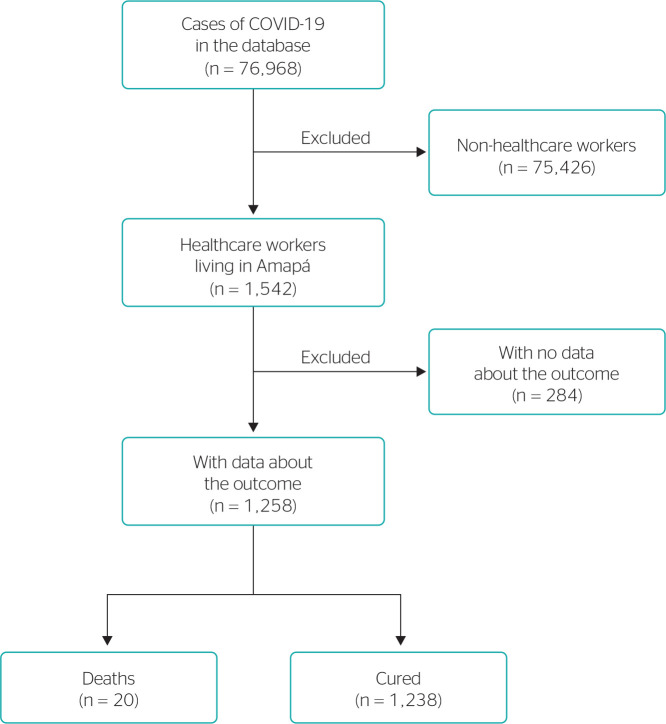
Flowchart of data analyzed in the study on deaths of healthcare workers due to COVID-19 in Amapá, Brazil, March 2020 to January 2021 (n = 1,258).

This study considered 1,542 healthcare workers living in Amapá of 76 968 cases of COVID-19 in the SESA database. Of these, 1258 (81.6%) had an outcome of death or cure due to COVID-19, thus eligible for analysis. The overall mortality rate was 1.59% (Figure 1).

Most of the 1,258 healthcare workers were women (67.7%; 852/1,258), multiracial (66.9%; 759/1,135), aged between 18 and 64 (98.3%; 1,226/1,247), and with no comorbidity (86.6%; 1,090/1,258). Most lived in the MMA (56.7%; 713/1,258) (Table 1). The mean age of the total healthcare workers was 40.3±10.42 years, and the median age was 39 years (interquartile range [IQR] = 14).

**Table 1:** Distribution of healthcare workers with COVID-19 according to clinical outcome of death or cure, according to sociodemographic characteristics and comorbidity, Amapá, Brazil, March 2020 to January 2021 (n = 1,258)

Variable (n)	Outcome		Total n (%)	p-value*
	Death n (%)	Cure n (%)		
Sex (n = 1,258)				
Male	10 (50.0)	396 (32.0)	406 (32.3)	0.0954^†^
Female	10 (50.0)	842 (68.0)	852 (67.7)	
Race/color (n = 1,135)^‡^				
Black	0 (0.0)	59 (5.3)	59 (5.2)	0.8038^§^
Yellow	1 (5.3)	110 (9.9)	111 (9.8)	
White	3 (15.8)	164 (14.7)	167 (14.7)	
Multiracial	14 (73.7)	745 (66.8)	759 (66.9)	
Indigenous	1 (5.3)	38 (4.3)	39 (3.4)	
Age group (years) (n = 1,247)^‡^				
18 to 64	16 (80.0)	1,210 (98.6)	1,226 (98.3)	0.0002^§^
65 or older	4 (20.0)	17 (1.4)	21 (1.7)	
Region of residence (n = 1,258)				
MMA	17 (85.0)	696 (56.2)	713 (56.7)	0.0109^†^
Inland	3 (15.0)	542 (43.8)	545 (43.3)	
Comorbidity (n = 1,258)				
Yes	9 (45.0)	159 (12.8)	168 (13.4)	0.0004^†^
No	11 (55.0)	1,079 (87.2)	1,090 (86.6)	

Source: Secretaria de Estado da Saúde do Amapá (Amapá Department of Health).

MMA = Macapá metropolitan area.

* p-value referring to chi-square test or Fisher’s exact test used to analyze association between the outcome and the independent variable.

† Chi-square of independence.

‡ Variable with missing information (race/color n = 123; age group n = 11).

§ Fisher’s exact test.

Among the 20 workers who died due to COVID-19, the majority were multiracial (73.7%; 14/19), aged between 18 and 64 (80%; 16/20) and with no comorbidity (55%; 11/20). Of the 20 deaths, 17 (85%) were healthcare workers living in the MMA. In addition, the number of deaths was the same as per sex (50% men 10/20; 50% women 10/20) (Table 1). The mean age of the cases of death was 49.95 years (standard deviation ±13.57), and the median age was 46.5 years (IQR = 16.75).

The variables “sex”, “age group”, “region of residence,” and “comorbidity” were chosen for logistic regression. In the multiple regression model, the following factors were associated with an increased chance of death among healthcare workers in Amapá: 65 years of age or older (vs. 64 years of age or younger) (OR = 10.43; 95%CI = 2.78-39.11), living in the MMA (vs. inland) (OR = 4.37; 95%CI = 1.25-15.29), and comorbidity (vs. no comorbidity) (OR = 4.52; 95%CI = 1.74-11.74). The model was adjusted according to the “sex” variable (Table 2). The C statistic of the model was 0.801 (95%CI = 0.709 0.891) and the p-value of the Hosmer-Lemeshow test was 0.287. Table 2 shows the results of the univariate and multiple logistic regression analyses.

**Table 2:** Logistic regression analysis to study the factors associated with the death of healthcare workers due to COVID-19, Amapá, Brazil, March 2020 to January 2021 (n = 1,258)

Variable (n)	Univariate regression			Multiple regression*		
	OR	95%CI	p-value	OR	95%CI	p-value
Sex (n = 1,258)						
Male	2.13	0.88-5.15	0.0946	2.12	0.84-5.33	0.1092
Female	1.00	-	-	1.00	-	-
Age group (years) (n = 1,247)^†^						
18 to 64	1.00	-	-	1.00	-	-
65 or older	17.79	5.38-58.82	0.0001	10.43	2.78-39.11	0.0005
Region of residence (n = 1,258)						
MMA	4.41	1.29-15.13	0.0182	4.37	1.25-15.29	0.0210
Inland	1.00	-	-	1.00	-	-
Comorbidity (n = 1,258)						
Yes	5.55	2.27-13.61	0.0002	4.52	1.74-11.74	0.0019
No	1.00	-	-	1.00	-	-

95%CI = 95% confidence interval; OR = odds ratio; MMA = Macapá metropolitan area.

## Discussion

This study calculated the mortality rate of COVID-19 among healthcare workers in Amapá, Brazil, and found the following factors associated with death: increasing age, comorbidity, and people living in the MMA.

As for the overall mortality rate, the data indicated a result higher than that of international studies that also analyzed deaths due to COVID-19 among healthcare workers, as observed in China (0.30%),^16^ the United States of America (0.61%),^17^ and Germany (0.20%).^18^ This may be due to a scenario of constraints these workers faced in Amapá, such as the lack of PPE that requires recycling after use, shortage of diagnostic tests, and poor medical follow-up of infected patients.^19^ Other factors may have influenced higher mortality rate in this study, such as false-negative cases due to insufficient accuracy of diagnostic tests.

Although the overall mortality rate found in this study was higher than in other similar studies,^16-18^ it was lower than that found in a previous study (2.36%)^20^ including the general population of Amapá. A lower mortality rate among healthcare workers was found abroad when comparing population studies, such as in Germany (0.20% vs. 4.60%, respectively)^18^ and in China (0.30% vs. 2.30%, respectively).^16^ Among the reasons for this difference are a) the average age of these workers is lower than the average age of the general population, since fatality and chances of death due to COVID-19 are higher in older people^2,20^ and b) broader access of healthcare workers to care and testing.^2^

Approximately 68% of the workers with COVID-19 in this study were women; higher than that found in a study carried out with the general population of Amapá, in which women represented 51.6% of the cases.^20^ This prevalence of women is relatively consistent with the results of Moscola et al.^1^ (73.6%) and Burrer et al.^17^ (73%) among North American healthcare workers, and Guerrero-Torres et al.^2^ (61.1%) in Mexico. This prevalence of SARS-CoV-2 infections among women may be partly explained due to the distribution of the sexes in the healthcare workforce. A World Health Organization (WHO)^21^ review of 104 countries estimated that women represent 67% of the healthcare workforce. Moreover, women are the majority among nursing professionals,^21^ who are constantly in contact with patients and play a key role in the pandemic, whose risk can collapse healthcare systems.^5,10^

Still regarding to sex, considered an important variable in the context of COVID-19, several studies have shown an association between men and death due to COVID-19.^2,22-24^ However, other studies have not found statistically significant increasing probability of death according to sex.^24^ Furthermore, regardless of statistical significance and confidence interval, the odds ratio for men (vs. women) has ranged 1.15 to 2.50 in most studies, according to the meta-analysis by Li et al.,^24^ which is in line with the data presented in this study. In this regard, due to the relevance of the variable “sex” in the current literature on COVID-19,^2,22-24^ we highlight the importance of adjusting the multiple regression model with this variable, even if it did not show statistical significance in the univariate regression.

This study showed that workers aged 65 years or older (vs. 18 to 64) had a higher chance of death due to COVID-19. Similar results were found in the study by Guerrero-Torres et al.,^2^ Cobre et al.,^23^ and Gómez-Belda et al.,^25^ whose multivariate analysis described higher chances of death in older individuals, whether they were healthcare workers or not. On the other hand, the results of Burrer et al.^17^ indicated a mortality rate 3.8 times higher in healthcare workers aged 65 years or older (10.3%), when compared to those aged 18 to 64 (2.7%). Among the possible reasons are the process of immunosenescence, cellular and molecular damage accumulated in vital systems, and higher prevalence of comorbidities. These factors, together or not, can limit the overall process of fighting against infections.^22,25,26^

Healthcare workers living in the MMA had a higher risk of death due to COVID-19. This area concentrates most of the population of Amapá and has relevant urban poverty and suburbanization: most residents live in overcrowded households and lack access to health services, basic sanitation, and transportation. In short, most of the population lives in neighborhoods with low levels of socioeconomic development.^11,14^ Studies have shown that suburbanization and low development are associated with a higher prevalence of COVID-19 and a longer gap between the onset of symptoms and diagnosis, which can increase the risk of death.^23,27^ Thus, it is worth noting that health services face challenges to deal with the pandemic in the MMA, thanks to insufficient medical-hospital structure and low density of healthcare workers per inhabitant, among other factors.^7,8,13^

Considering the high demographic density of the MMA when compared to other inland municipalities,^11^ it should be noted that the most populated municipalities were proportionally less affected in the early outbreak of SARS-CoV-2. However, this relationship reversed during the pandemic.^28^ Ribeiro et al.^28^ analyzed data from Brazilian municipalities until August 2020 and found higher prevalence and mortality rate due to COVID-19 in the long term in more populated municipalities. One of the reasons is higher social and economic interaction in large and medium-sized cities when compared with small towns.^28^ In addition, it is noteworthy that the overall mortality rate of the general population in the MMA (1.43 deaths/1,000 inhabitants) was approximately 65% higher than that in the inland (0.86 deaths/1,000 inhabitants) until the 12th epidemiological week of 2021.^11,13^ This scenario and evidence may support the result of a higher chance of death among healthcare workers living in the MMA.

Moreover, the MMA concentrates major medical and hospital health services in Amapá.^8,11^ Thus, the number of professionals exposed to high viral loads is much higher in the MMA than in the inland. Exposure to high viral loads related to work in specific sectors, such as intensive care units, may represent greater risks of infection and possible worsening of the disease.^3,4,11^

This study showed another individual factor that increased the chance of death due to COVID-19. The presence of comorbidity had a result similar to that found in another study with healthcare workers carried out in Mexico, in which the chance of death ranged 1.26 (95%CI = 1.18-1.34) (one comorbidity) to 1.47 (95%CI = 1.37-1.58) (two comorbidities or more) when compared to the group with no comorbidities.^2^ Higher mortality rates in individuals with comorbidities were also identified in studies on the general population.^16,20,22^

It is worth noting that the effect of a comorbidity, such as diabetes or hypertension, may be different as per age group.^29^ In younger adults, diabetes mellitus stands out as one of the comorbidities with the greatest effect as a single risk factor of death due to COVID-19.^29^ These two individual factors, increasing age and clinical comorbidity, may act together or interactively for a greater chance of death due to COVID-19. Thus, due to higher prevalence of chronic endocrine and cardiovascular diseases in elderly individuals, studies have pointed out some difficulty to determine the real influence of each variable on the outcome.^22,30^ For example, in the case of diabetes mellitus, the risk of death due to COVID-19 may be confounded by increasing age and hypertension, while the risk relationship for increasing age and hypertension may be dependent on each other.^29^ This study also identified potential confounding between these factors, thus agreeing with the literature.

This study analyzed the official population data of Amapá. Underreporting of cases of COVID-19, incomplete notification, and possible flaws in the accuracy of diagnostic tests, especially serological tests, may be factors that partly affect the external validity of this study. The outcome measurement bias was minimized by considering an objective outcome and excluding active cases of COVID-19. However, even though the multiple model of this study showed good calibration and fit to the data, it is worth noting as a limitation for analysis the restricted number of independent variables available in the database, especially about the occupation of professionals, workplace, date of onset of signs or symptoms and date/location of death, as well as the reduced number of events of interest.

## Conclusions

The place of living and individual factors, such as increasing age and comorbidity were associated with a greater risk of death due to COVID-19 among healthcare workers in Amapá. Thus, considering that a high number of sick leaves and deaths of healthcare workers due to COVID-19 can compromise the local health system, government policies are needed to protect healthcare workers in risk groups by switching to a less exposed role, teleworking (ensuring wage and other labor rights), as well as adequate provision of PPE, given the high risk of having the severe form of COVID-19.
